# Participation of Acoustic and Electric Hearing in Perceiving Musical Sounds

**DOI:** 10.3389/fnins.2021.558421

**Published:** 2021-05-05

**Authors:** Sonia Duret, Emmanuel Bigand, Caroline Guigou, Nicolas Marty, Philippe Lalitte, Alexis Bozorg Grayeli

**Affiliations:** ^1^Otolaryngology Department, Dijon University Hospital, Dijon, France; ^2^LEAD Research Laboratory, CNRS UMR-5022, Bourgogne-Franche-Comté University, Dijon, France; ^3^ImVia Research Laboratory, Bourgogne-Franche-Comté University, Dijon, France; ^4^Institut de Recherche en Musicologie (IReMus), CNRS- UMR 8223, Bourgogne-Franche-Comté University, Dijon, France

**Keywords:** bimodal hearing, cochlear implant, musical perception, musical test, quality of life

## Abstract

**Introduction:** The objective of our study was to evaluate musical perception and its relation to the quality of life in patients with bimodal binaural auditory stimulation.

**Materials and Methods:** Nineteen adult patients with a cochlear implant (CI) for minimum 6 months, and moderate to severe contralateral hearing loss with a hearing aid (HA), and 21 normal hearing adults were included in this prospective, cross-sectional study. Pure-tone and speech audiometry, musical test evaluating sound perception characteristics and musical listening abilities, Munich questionnaire for musical habits, and the APHAB questionnaire were recoded. Performance in musical perception test with HA, CI, and HA + CI, and potential correlations between music test, audiometry and questionnaires were investigated.

**Results:** Bimodal stimulation improved musical perception in several features (sound brightness, roughness, and clarity) in comparison to unimodal hearing, but CI did not add to HA performances in texture, polyphony or musical emotion and even appeared to interfere negatively in pitch perception with HA. Musical perception performances (sound clarity, instrument recognition) appeared to be correlated to hearing-related quality of life (APHAB RV and EC subdomains) but not with speech performances suggesting that the exploration of musical perception complements speech understanding evaluation to better describe every-day life hearing handicap.

**Conclusion:** Testing musical sound perception provides important information on hearing performances as a complement to speech audiometry and appears to be related to hearing-related quality of life.

## Introduction

Binaural hearing is essential in everyday life since it reduces head-shadow effect, enhances speech discrimination in noise and provides sound localization capabilities (Avan et al., 2015). Binaural hearing can be achieved in patients with cochlear implants (CI) and a contralateral hearing, with or without hearing aid (HA). The combined use of acoustic auditory stimulation and electrical stimulation is often called bimodal hearing. Although this situation does not restitute stereophony, several studies have shown that patients combine electrical stimulation with contralateral acoustic amplification to enjoy binaural functions to some extent (Morera et al., 2005; Ching et al., 2007; Firszt et al., 2012; Illg et al., 2014; Van Loon et al., 2017; Vroegop et al., 2018). These studies report an improvement in the perception of speech in both silent and noisy contexts together with improved sound quality and music perception (Kong et al., 2005).

In an implanted cochlea, the number of functional channels (stimulated nerve endings producing a distinctive pitch) are dramatically reduced in comparison to a normal ear, and each electrode codes for a large frequency band (Jay, 2004). Hence, CI depletes the complex sounds from their spectral cues. The lack of frequency discrimination combined to the impossibility of timbre recognition degrade the sound quality with a CI (Jay, 2004). Disposing of a higher number of electrodes in modern CIs has improved the frequency discrimination but increasing resolution by this mean quickly reaches physiological limits (Jay, 2004). Simultaneous stimulation of several electrodes to code for complex sounds can lead to current summation, a reduction in the spatial selectivity of neural excitation, and finally, to a decrease in sound quality (McDermott et al., 2003). The fine temporal structure also plays an important role in the quality of sound perception. This parameter corresponds to minute variations in amplitude and frequency of the sound waves ranging from 0.6 to 10 kHz. It is the basic ingredient in several sound attributes such as pitch and timbre, and also influences source location and even loudness (Rosen, 1992). Coding the temporal fine structure in CI by delivering the fluctuations of the electrical signal to the cochlea provides both temporal and spatial information. Theoretically, it increases speech perception in background noise and timbre recognition (Todd et al., 2019). However, this type of coding algorithm would require large number of functional channels and a very rapid rate of stimulation, creating again undesired channel interaction.

While CI is effective for speech perception (Gaylor et al., 2013), the quality of music perception is generally poor and highly variable between patients (Brockmeier et al., 2011; Drennan et al., 2015; Prevoteau et al., 2018; Riley et al., 2018). This issue has to be addressed since music is a crucial aspect of hearing: it can be used in auditory and cognitive rehabilitation (Driscoll, 2012; Van Besouw et al., 2014; Hutter et al., 2015; Smith et al., 2017) and is a significant aspect for the quality of life (Lassaletta et al., 2007; Dritsakis et al., 2017a, b). Patients’ musical habits tend to change after cochlear implantation: even though half of patients still declare enjoying music, they spend less time listening to it (Lassaletta et al., 2007). Numerous studies have explored musical perception with CI (Brockmeier et al., 2011; Limb and Rubinstein, 2012; Buyens et al., 2014; Drennan et al., 2015; Nemer et al., 2017; Choi et al., 2018; Prevoteau et al., 2018; Riley et al., 2018). Most works have studied the perception of its spectral (pitch, melody, and sound harmony) and temporal characteristics (rhythm and tempo). Although implantees perform as well as normal-hearing individuals for detecting temporal differences, they have more difficulty with spectral characteristics (Brockmeier et al., 2011; Choi et al., 2018; Prevoteau et al., 2018). A slight difference in tone is difficult to perceive (Brockmeier et al., 2011; Choi et al., 2018; Prevoteau et al., 2018; Riley et al., 2018), and simple harmonies are preferred (Nemer et al., 2017; Riley et al., 2018). Patients also prefer music with a dominant rhythmic line (bass, percussion), simplified harmonies and songs (Buyens et al., 2014; Riley et al., 2018). Bimodal hearing stimulation with a CI combined to a contralateral HA has been shown to improve not only the quality of perceived musical sound (Cheng et al., 2018), but also its emotional aspect (Shirvani et al., 2016).

The link between musical perception and quality of life has been investigated through validated questionnaires. Although quality of life is significantly improved after implantation, the quality of musical perception is generally degraded. At the same time, patients with better musical perception are found to have a better quality of life (Lassaletta et al., 2007; Dritsakis et al., 2017a, b). However, the musical parameters which contribute to the quality of hearing remain unclear in cochlear implantees.

The objective of our study was to further analyze the contribution of acoustic and electric hearing to different aspects of musical perception and their possible relation to the quality of life.

## Materials and Methods

### Population

We conducted a prospective cross-sectional study on patients with unilateral profound hearing loss implanted with a CI for more than 6 months and a moderate (pure-tone average 500–4,000 Hz, PTA 41–70 dB) to severe (71–90 dB) contralateral hearing loss (International Bureau for Audiophonology, 2020) fitted with a HA. All patients had a significant hearing gain with their aid, with an aided PTA < 60 dB measured by free-field audiometry. We excluded patients with a normal contralateral ear.

We collected patient data including duration of profound hearing loss, etiology of hearing loss, age at implantation and CI brand. Audiometric data were also collected (free-field speech and pure-tone audiometry with and without the HA and with and without the CI, including the speech reception threshold, SRT and word discrimination score, WDS with and without lip reading after implantation). When SRT could not be reached, level of maximum word score (dB) was indicated. The study received the approval of the local ethical research committee (CPP Est III). An informed consent was signed by all patients.

Thirty-six patients currently followed-up in our center and who met these criteria were contacted. Among these patients, 17 (47%) declined to participate in the study. Finally, 19 adult patients were included. The group was composed of 12 women and seven men. Their average age was 61.2 ± 4.02 years (range: 19–78). Among patients, the hearing loss etiology was advanced otosclerosis in four (21%), sudden deafness in three (16%), congenital deafness in three (16%), Meniere’s diseases in two (10%), toxic in one (5%), and unknown in six (32%). The duration of profound deafness was 10 ± 2.8 years (range: 0–45), and the time between implantation and inclusion was 32 ± 6.5 months (range: 6–110). The mean duration of hearing aid usage was 29 ± 4.3 years (range: 3–56).

Patients were equipped with Cochlear® CI 522® implant and Nucleus 6® processor in seven cases (37%), Advanced bionics® HiRes Ultra® implant and Naida CI Q90® processor in four (21%), Oticon Medical® Neuro ZTI® implant and Neuro 2® sound processor in four (21%), and Medel® Synchrony® implant and Sonnet® sound processor in four (21%) patients. Contralateral hearing aids were systematically reviewed for optimization within the 3 months period before inclusion. This included a technical check-up by the audiologist to verify that the hearing aid is correctly functioning, a free-field audiometry with the evaluation of the audiometric gain, and eventual fitting.

A control group of 21 adults was also tested. This group comprised eight men, and 13 women with a normal hearing tested by pure-tone audiometry. The mean age in this group was 60.9 ± 4.14 years (range: 19–88).

### Musical Perception Test

The ‘‘BbMAT’’ musical perception test, (Burgundy best Musical Aptitude Test^1^) includes three main categories: sound, syntax and music sense. Each category includes four different test series, each with three subtests for the sound and syntax categories and two subtests for the music sense category. In this test material, we selected eight trials with a level of difficulty that could be adapted to CI patients. The sound material was prepared from musical samples that were systematically modified with a signal processing software (Audiosculpt, Ircam software, Paris, France). A laptop interface provided the information on the screen, an example for each test, and the response buttons on which the participant had to click in order to register his/her response. The required response was a forced choice between two propositions or a selection among a list (instruments). Each sound sample lasted 10 s. and could be listened *ad lib*. The recordings were played in an audiometric booth, by two loudspeakers (Planet M, Elipson, Champigny-Sur-Marne, France) placed frontally at 1 m in a silent environment. The sound level was adjusted to an intensity considered comfortable for the patient, defined before the test. The test was performed once with the HA alone, a second time with the CI alone (contralateral ear masked with white noise at PTA + 20 dB), and with the HA + CI. The learning effect was minimized by hiding the responses from the patient. Test order was not randomized because test items concerned unrelated sound or music qualities. Exposing the subject to one test could not enhance the performance to other items. Moreover, items were not compared to one another. The trial was short (approximately 30 min.) to avoid the fatigue effect. Score for each trial was recalculated to provide a mark out of 10.

#### Sound Characteristics Category

This category included a pitch perception trial (six tests) where exercises consisted in distinguishing between two risings or falling notes sang by a professional female singer (minor and major sixth intervals). The instrument recognition trial included nine tests with musical excerpts where the patient had to recognize which instrument was playing from a list of nine illustrated instruments (harpsichord, cello, acoustic, guitar, trumpet, clarinet, flute, marimba, djembe drum, and marching snare). The timbre recognition trial included sound brightness, roughness, and clarity with six tests for each characteristic. In each test two samples were compared, and participants had to indicate which sample was brighter, rougher or more clear (sharper attack).

The latter trial focused on three sound dimensions (Wallmark and Kendall, 2018):

- Brightness pertains to the spectral envelope of the sound. High energy in high-frequency spectrum is defined by a bright sound.
- The roughness corresponds to the spectral flux (spectrum instability in time). Higher flux corresponds to a rougher sound.
- The clarity is related to the attack curve of the sound. A direct attack causes a steep slope of amplitude in the temporal envelope and a clear sound.

#### Syntax and Music Sense Categories

We evaluated the capacity to recognize musical lines in the same sound sample (polyphony). The subject had to indicate whether one or more instruments were playing at the same time (eight tests). We also evaluated the perception of sound texture in a trial. A sound sample was presented, and the examinee had to choose between “dense” or “airy sound” (six tests).

In the music sense trial (six tests), we tested the emotional information provided by the music. The participant had to recognize the emotion reflected by selecting between “calm,” “fear,” and “anger.” In addition, a commentary box was provided to type general impressions for this task.

### Munich Music Questionnaire

Patients completed a questionnaire on musical habits, specifically the Munich music questionnaire (MMQ) developed by S. J. Brockmeier (Brockmeier et al., 2007). It includes questions on musical experience ranging from listening time to sound quality, instrument recognition, and past and present importance and involvement in music.

### APHAB Quality of Life Questionnaire

We also asked participants to complete a quality of life questionnaire, APHAB (Abbreviated Profile of Hearing Aid Benefit). It includes 24 questions about different everyday life situations on hearing comfort. They are divided into four categories:

- Ease of communication (EC): effort to communicate under relatively favorable conditions.
- Reverberation (RV): communication in rooms with high reverberation.
- Background noise (BN): communication in environments with high background noise.
- Aversiveness (AV): unpleasant or disturbing aspect of the surrounding sounds.

For each question, the patient had to quantify the frequency at which he or she was exposed to the situation. Each frequency corresponds to a percentage: always (99%), almost always (87%), generally (75%), half the time (50%), sometimes (25%), rarely (12%), and never (1%). It reflects the percentage of difficulties experienced: the lower the percentage, the better the hearing comfort. The percentages were then grouped by category to calculate the average score for ease of communication, reverberation, background noise and aversiveness. Each item was evaluated before and after cochlear implantation.

### Statistical Analyses

Graphpad Prism software (v.6, Graphpad Inc., San Diego, CA, United States) was used for statistics. The quantitative descriptive variables were described as means and standard error of the mean (SEM), and the qualitative descriptive variables by frequencies and percentages. The music test scores did not have a normal distribution for all test items (D’Agostino and Pearson’s test). Consequently, a non-parametric Wilcoxon signed-rank test was employed to compare scores vs. random level. This was followed by a Bonferroni correction for multiple comparisons, and the adjusted *p*-values based on familywise error rates were reported. To compare different hearing conditions for each test item, a non-parametric Kruskal–Wallis test was used. This analysis was corrected for multiple comparisons by Dunn’s method and adjusted *p*-values were reported. APHAB scores passed D’Agostino and Pearson’s normality test. A 2-way ANOVA followed by Sidak’s multiple comparisons test was conducted and adjusted *p*-values are reported. The global APHAB scores were evaluated by a paired *t*-test.

## Results

### Hearing Performance

**FIGURE 1:**
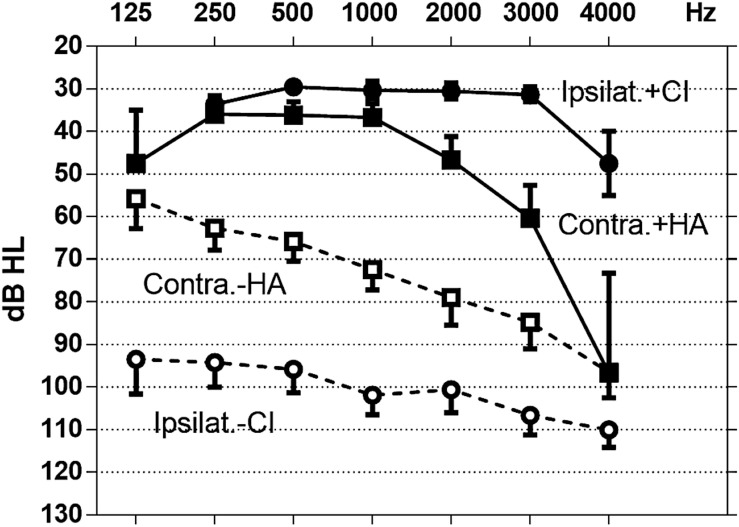
Free-field pure tone audiometry in patients. Ipsilateral ear with (+CI) and without (−CI) cochlear implant and contralateral ear with (+HA) and without (−HA) hearing aid were tested separately. Values represent mean ± standard error of mean.

The ipsilateral PTA before and after implantation were 101 ± 4.8 dB and 30 ± 1.5 dB, respectively. For the contralateral ear, the PTA without and with the HA were measured at 76 ± 5.3 dB and 45 ± 3.7 dB respectively (Figure 1). With the CI, the ipsilateral SRT reached 54 ± 6.4 dB (range: 20–120) without lipreading and 42 ± 7.6 dB (range: 20–120) with lip reading. Ipsilateral WDS with CI was estimated as 67 ± 7.59% (range: 0–100) without lipreading and 83 ± 6.26% (range: 20–100) with lipreading. On the aided contralateral side, SRT was measured at 41 ± 2.3 dB (range: 25–55), and the WDS was 85 ± 3.8% (range: 50–100).

### Musical Performance

**FIGURE 2:**
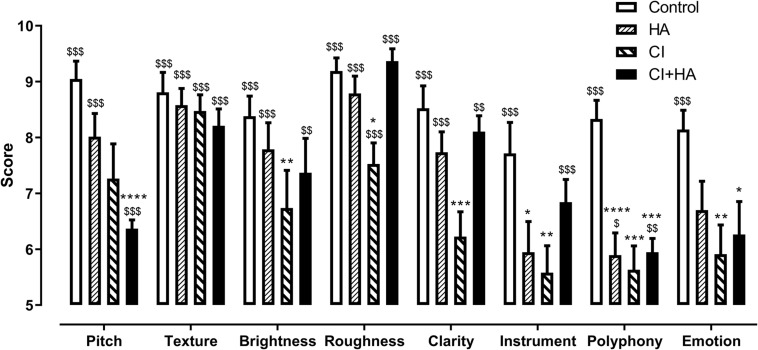
Musical test results. Controls (*n* = 21, white) and Patients (*n* = 19, hatched or black) were evaluated by “Burgundy best Musical Aptitude Test” evaluation several musical aspects (horizontal axis). Scores were converted to marks out of 10. Values are represented as means ± standard error of mean. Patients took the test with hearing aids only (HA), cochlear implants only (CI) and hearing aid plus cochlear implant (HA + CI). Adjusted *p*-values: ^$$$^*p* < 0.0001, ^$$^*p* < 0.01, and ^$^*p* < 0.05 vs. random level (Firszt et al., 2012), Wilcoxon signed-rank test with Bonferroni correction for multiple comparisons. ****p < 0.0001, ***p < 0.001, **p < 0.01, and *p < 0.05, vs. control for each test item, £: *p* < 0.05 vs. HA + CI of the same item, Kruskal–Wallis test with Dunn’s correction for multiple comparisons.

All normal subjects and CI participants completed the test in approximately 30 min. All items could be clearly understood, and the instructions could be followed easily (Figure 2). Controls obtained better overall scores than patients with the HA alone, the CI alone and HA + CI (8.5 ± 0.13, *n* = 168 for controls vs. 7.4 ± 0.17, 6.7 ± 0.18, and 7.3 ± 0.16, for patients respectively, *n* = 152, *p* < 0.0001, Kruskal–Wallis test followed by Dunn’s correction). For the overall performance, patients scored higher with HA than with CI alone (*p* < 0.01 Friedman test followed by Dunn’s correction). Nevertheless, CI + HA performed better than the implant alone (*P* < 0.01, Friedman test followed by Dunn’s correction, *p* < 0.01 Figure 2), suggesting that bimodality compensated for the CI deficit for musical perception.

The combined prostheses performed better than HA or CI alone for brightness, roughness, clarity, and instrument recognition (Figure 2). It is noteworthy that patients with CI + HA performed as well as controls for roughness. In the remaining categories, i.e., texture, polyphony, and emotion, combination of HA + CI did not compensate for the lower performance of CI. For pitch perception, CI + HA further degraded the performance with HA alone (Figure 2).

For emotions, controls performed better than patients. While patients could categorize above random level with HA only, the scores dropped to random level with CI or HA + CI suggesting a negative interference between HA and CI for this task (Figure 2). In their comments, six patients reported that they found the test subjective, particularly for the notions of fear and anger, even after the answers were revealed. These patients reported feeling fear when hearing the music used for anger and vice versa. This difficulty was not encountered in controls.

### APHAB Quality of Life Questionnaire

**FIGURE 3:**
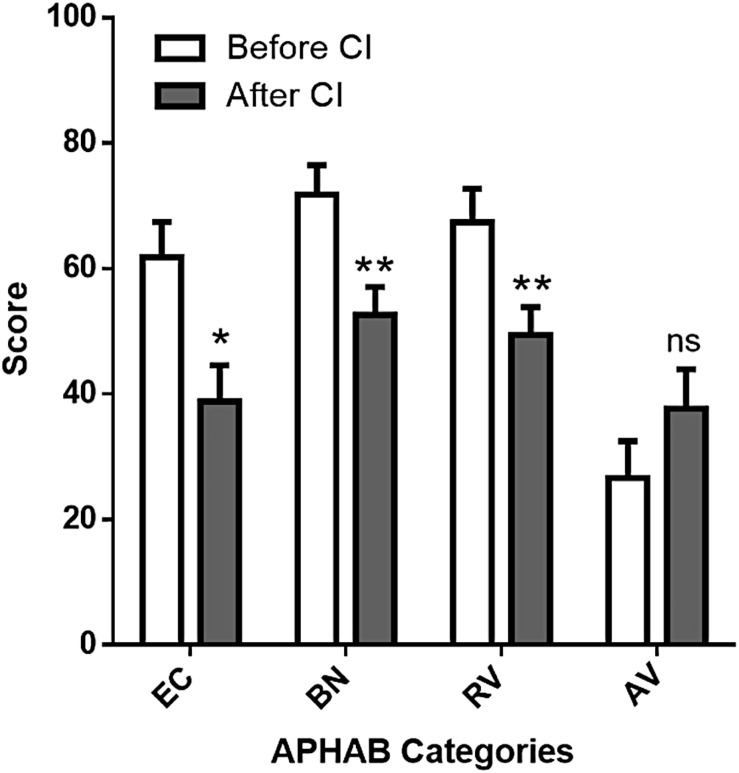
APHAB scores before and after cochlear implantation. Values are represented as mean ± standard error of mean for each subdomain: ease of communication (EC), background noise (BN), reverberation (RV), aversiveness (AV). Lower scores indicate better functional results. ns, not significant. *Adjusted *p*-value < 0.05, two-way ANOVA followed by a Sidak’s test for multiple comparison.

Cochlear implantation appeared to provide good results for ease of communication (EC, 61.76 ± 5.62% before the CI and 38.79 ± 5.77% after CI, *n* = 19, *p* < 0.05, ANOVA followed by Sidak’s posttest, Figure 3). Situations with background noise (BN) seemed also improved (71.74 ± 4.74% before vs. 52.59 ± 4.44% after CI, *p* < 0.05, ANOVA followed by Sidak’s posttest, Figure 3). In contrast, scores in reverberation (RV) and aversiveness (AV) subdomains did not vary significantly (Figure 3). The global score improved significantly from 67 ± 4.6% to 47 ± 4.3% (*p* < 0.05, paired *t*-test) with an average gain of 20 ± 6.1%.

### Munich Music Questionnaire

The average score on the importance of music in every-day life was moderate (score: 3.2 ± 0.33, range: 1–5). Nevertheless, thirteen (68%) trained themselves by listening to already known music, and 11 (58%) regularly attended musical events. Almost all patients reported perceiving pleasant sounds (*n* = 18, 94%). Sixteen patients (84%) declared perceiving melodies well and 17 (89%) declared a good perception of rhythms. Five patients (26%) had previously followed musical courses, and six (32%) had previously played a musical instrument. Two of them continued after CI.

Participants mainly listened to pop music after implantation (pop: 100%, rock:26%, classical; 21%, opera: 21%, religious, folk, rap, and techno: 5% each). There was no change in musical style before and after implantation (data not shown). Most enjoyed listening to both solo and orchestral instruments (*n* = 12, 63%).

The MMQ questionnaire also included self-assessment items on musical performance. Seven patients (37%) reported being able to recognize a wrong note and six (32%) were able to find a rhythmic error or compare performances. Only two (11%) felt that they could sing in tune and would sing in public. Remaining results of the questionnaire pertaining to musical activities before and after CI do not show a significant change (Table 1).

**TABLE 1:** Munich musical questionnaire before and after cochlear implantation (CI).

	**Before CI**	**After CI**
**How often do you listen to music?**		
Often	3 (16)	6 (32)
Sometimes	12 (63)	10 (53)
Never	4 (21)	3 (16)
**How long did you listen to music each day?**		
<30 min	11 (58)	7 (37)
30 min–1 h	6 (32)	4 (21)
1 h–2 h	1 (5)	3 (16)
>2 h	0	5 (26)
All day long	1 (5)	0
**Do you currently play or have you played an instrument?**		
Often	1 (5)	1 (5)
Sometimes	1 (5)	1 (5)
Never	17 (89)	17 (89)
**Did you sing or do you sing?**		
Often	2 (11)	2 (11)
Sometimes	4 (21)	3 (16)
Never	13 (68)	14 (74)

*Figures indicate n (percentage) of positive responses.*

### Relationship Between Questionnaires, Musical and Auditory Performances

**FIGURE 4:**
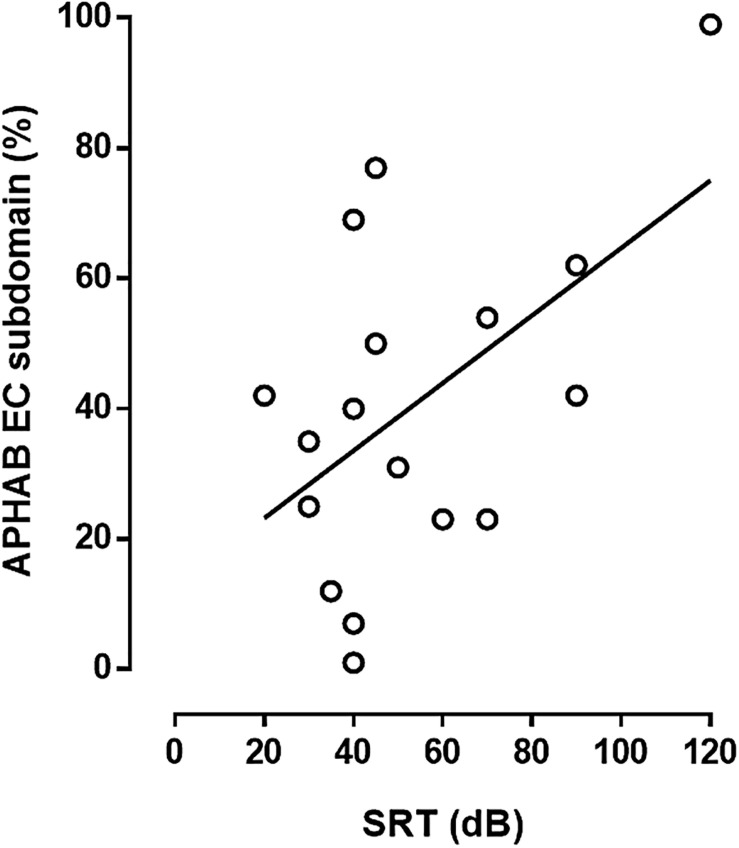
Relation between auditory performances and APHAB score. Speech reception threshold (SRT) with CI and without lipreading was correlated to ease of communication (EC,%) subdomain of APHAB after CI (*Y* = 0.5X + 12.8, *R* = 0.56, *n* = 17, *p* < 0.05, *F*-test).

**FIGURE 5:**
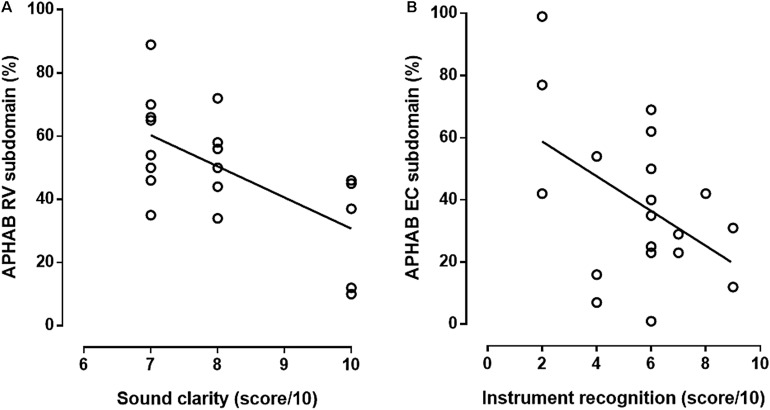
Relation between musical performances and APHAB score. **(A)** Sound clarity with hearing aid combined to cochlear implant was correlated to reverberation (RV,%) subdomain of APHAB (*Y* = –9.8X + 123, *R* = 0.63, *n* = 19, *p* < 0.01, *F*-test). **(B)** Recognition of instruments with cochlear implant was correlated to ease of communication (EC,%) subdomain of APHAB after CI (*Y* = –5.59X + 70.0; *R* = 0.49, *n* = 19, *p* < 0.05, *F*-test).

Auditory performance, as evaluated by SRT in CI + HA condition, was correlated to the APHAB EC subdomain score (Figure 4). Musical performances also appeared to be related to APHAB questionnaire results. Indeed, sound clarity score in HA + CI condition was negatively correlated to RV subdomain score after rehabilitation (Figure 5A). There was no correlation between sound clarity scores in CI or HA condition and RV scores, suggesting the contribution of bimodal binaural hearing in reverberating conditions. Interestingly, we observed a correlation between the instrument recognition score with CI and the ease of communication (Figure 5B).

We did not observe a relationship between frequency of musical education or music listening and musical test performances (data not shown) suggesting that hearing performance and enjoyment may not be directly related.

In addition, speech audiometry and performances on musical perception test were not correlated (data not shown). This suggested that musical perception test explores a different domain and may be complementary to speech audiometry in assessing performance in daily situations.

## Discussion

We developed a musical test battery which explored musical perception capabilities at several levels ranging from sound characteristics to evoked musical emotions. We showed that this battery was applicable to normal hearing and CI patients without any ceiling or floor effect. The tests discriminated normal subjects from hearing-impaired participants. They could also show the effect of hearing rehabilitation mode. Bimodal stimulation improved musical perception in several categories such as sound brightness, roughness and clarity, but CI did not add to HA performances in texture, polyphony or musical emotion and even appeared to interfere negatively in pitch perception. Musical auditory performances appeared to be related to the quality of life as evaluated by APHAB but not with speech performances suggesting that exploration of musical perception potentially complement the conventional speech audiometry to better describe the every-day life handicap.

The performance of a hearing rehabilitation system is basically judged by audibility, speech discrimination and sound quality (Boretzki, 1999). While the first two parameters can be easily evaluated by robust psychophysical methods, the latter is more complex to comprehend by the patient and the audiologist through an analytic description (Devocht et al., 2017). In this field, the main question is whether sound quality attributes in hearing impaired individuals are comparable between subjects or if each patient constitutes an individual reference (Boretzki, 1999). Using a descriptor panel to analyze sound quality and asking the patient to rate each feature is feasible in patients with HA and provides coherent results. However, a large intra individual discrepancy in the ratings is observed and suggests that there is no stable reference for quality attributes and even worse, which the standard varies during the test (Boretzki, 1999). The absence of a consistent base line for many of these features may also explain the significant inter individual discrepancy (Devocht et al., 2017). Consequently, this type of method potentially limits comparisons in patients (Devocht et al., 2017) and does not allow to assess performances. To overcome this limitation, we proposed a categorization task instead of a semi-quantitative appreciation and we provided the patients with an example at the beginning of each test. The categorization provides the baseline and potentially tends to reduce the variance.

Auditory categorization requires the detection of details, and at the same time, generalization across variants in the same group (Liu et al., 2019). This contradictory function is not limited to the auditory system but is largely applied to other sensory inputs, especially for visual information (e.g., face reconnaissance, 37). In humans, face classification is very efficient and based on the analysis of only a small number of intermediate-level features (e.g., combination eyes and nose) by the visual cortex (Ullman et al., 2002). A recent study showed that this type of processing can be generalized to auditory inputs and across animal species (Ullman et al., 2002). In marmosets, a small number of mid-level features (sequences of twitters, phees, and trills with characteristic bandwidth and temporal integration period) allow a high-performance classification of calls. At the neuronal level, this function is insured by feature-specific neurons in the primary auditory cortex (Ullman et al., 2002). Although the sound classification mechanisms in cochlear implantees have not been specifically studied, the use of mid-level features for categorization of sound samples is compatible with relatively high scores obtained in CI-only condition, especially for texture.

Including the pitch perception in our test battery allowed us to verify a basic, yet consistent, auditory parameter in controls and implantees. Counter intuitively, patients had a poorer pitch perception with HA + CI than with HA or CI separately. This observation suggested a negative interference between HA and CI for the task of discrimination between ascending and descending notes. It could be explained by the altered pitch place function and degraded temporal cues in the implanted ear (Jay, 2004). The lack of frequency discrimination further degrades the quality of sound perception. Indeed, the median pitch difference that implantees could discriminate is five tones (Brockmeier et al., 2011). We hypothesize that the discrepancy of interval perception between the ears alters the scores in binaural condition. Such negative interference in bimodal hearing has been related the mismatch between the pitch perceived by the CI and contralateral residual hearing in low and medium frequencies. Indeed, when CI electrodes coding for the same frequencies as those provided by the contralateral functional hearing are inactivated patients with bilateral and bimodal hearing perceive a more natural sound (Richard et al., 2012). Negative interference of CI on HA performances highlights the difficulty of finding the best possible match between acoustic and electric hearing inputs in binaural patients (Keilmann et al., 2009). Recent studies on bimodal pitch perception offer another argument for the poor performance in classifying ascending and descending notes (Hartling et al., 2020; Oh and Reiss, 2020). In patients with a hearing loss rehabilitated by HA or CI, a broad binaural pitch fusion is generally observed (Hartling et al., 2020; Oh and Reiss, 2020). Sounds differing in pitch by as much as three to four octaves are perceptually integrated across ears. This phenomenon is subject to great inter individual variability both in adults (Oh and Reiss, 2020) and children (Hartling et al., 2020), depending on the mode of rehabilitation, the degree of loss and the duration of hearing deprivation. This phenomenon can mask the interval between the two presented notes by reducing the frequency resolution of the ear with a HA to the level of the contralateral CI in bimodal patients.

The influence of cultural background cannot be eliminated in tests based on music. The performance was homogeneous in the controls suggesting that the effect of musical education was limited in this group. However, patients with musical education had higher pitch perception scores than those with no musical background. This observation is consistent with the literature showing the influence of culture on performances in musical and auditory activities (Lee and Hung, 2008). Regardless of language skills, musicians score higher than non-musicians in tone identification in mandarin Chinese (Lee and Hung, 2008). Inversely, speaking a tonal language seems to positively influence the perception of musical pitch. A group of Chinese-speaking adults performed better in musical pitch perception when compared to a group speaking an atonal language (French-speaking and English-speaking Canadians (Wong et al., 2012). Similar observations were reported in Chinese-speaking patients with amusia vs. those speaking an atonal language (Bidelman et al., 2011). During the test elaboration, we minimized the cultural influence by avoiding cultural references to instruments or melodies. No prior knowledge was required to understand the tests. The selected instruments are widely used across the world. Each series began with an example. Overall, the test was based more on musical sounds than on composed music.

Patients performed poorly for the categorization of elicited emotions. While the test distinguished normal-hearing subjects from the patients, it did not reveal an improvement of emotion scores by adding the HA to CI. This may appear contradictory to what other investigators have shown in children with bimodal rehabilitation in comparison to CI alone for the categorization of sad vs. happy music based on tempo and mode (Shirvani et al., 2016). This contrast may be explained by the study protocol (e.g., type of emotion elicited, auditory cues such as rhythm and musical mode, number of categories). Pitch-fusion, and negative HA-CI interference may also contribute to the poor performance in HA + CI condition.

Despite the well-known deterioration of sound perception by the CI (Shirvani et al., 2016; Devocht et al., 2017), our bimodal patients did not change their musical habits after implantation (e.g., style of music, duration of listening). In accordance with other series (Drennan et al., 2015), the absence of correlation between listening time and performance suggests that musical enjoyment and performance are not directly related, and questionnaires such as MMQ provide limited information on the latter.

The heterogeneity of CI coding strategies and sound processing algorithms as well as HA fitting could have masked some effects in this population. Nevertheless, score variability was moderate in each item and consistent within the hearing condition (CI, HA and HA + CI). Further tests, especially in patients with single-sided deafness and CI, will potentially reduce this heterogeneity and provide precious data on ipsilateral vs. contralateral performances and, also the binaural interactions. The effect of specific CI or HA fittings can also be evaluated by this test battery.

In conclusion, BbMAT music test battery yielded data on perceived sound quality which was consistent with hearing-related quality of life and previous studies. Contralateral HA seemed to complete the auditory cues provided by the CI and brought the musical performance of the implanted patients closer to normal. BbMAT could be used to evaluate hearing in a new analytical way and as a complement to conventional audiometry.

## Data Availability Statement

The raw data supporting the conclusions of this article will be made available by the authors, without undue reservation.

## Ethics Statement

The studies involving human participants were reviewed and approved by CCP EST III. The patients/participants provided their written informed consent to participate in this study.

## Author Contributions

SD, EB, PL, and ABG designed the study. PL and NM developed the tests. SD included the participants and conducted the tests. SD, EB, CG, and ABG analyzed the data and prepared the manuscript. All authors contributed to the article and approved the submitted version.

## Conflict of Interest

The authors declare that the research was conducted in the absence of any commercial or financial relationships that could be construed as a potential conflict of interest.

## Footnotes

1 http://leadserv.u-bourgogne.fr/~cimus/
